# Identification and In Silico Characterization of a Novel *COLGALT2* Gene Variant in a Child with Mucosal Rectal Prolapse

**DOI:** 10.3390/ijms23073670

**Published:** 2022-03-27

**Authors:** Anna Sadakierska-Chudy, Paweł Szymanowski, Arleta Lebioda, Rafał Płoski

**Affiliations:** 1 Department of Genetics, Faculty of Medicine and Health Sciences, Andrzej Frycz Modrzewski Krakow University, Gustawa Herlinga-Grudzinskiego 1, 30-705 Krakow, Poland; 2 Department of Gynecology and Obsterics, Faculty of Medicine and Health Sciences, Andrzej Frycz Modrzewski Krakow University, Gustawa Herlinga-Grudzinskiego 1, 30-705 Krakow, Poland; pszymanowski@afm.edu.pl; 3 Department of Molecular Techniques, Department of Forensic Medicine, Wroclaw Medical University, M. Curie-Sklodowskiej 52, 50-369 Wroclaw, Poland; arleta.lebioda@umw.edu.pl; 4 Department of Medical Genetics, Warsaw Medical University, Pawinskiego 3c, 02-106 Warsaw, Poland; rploski@wp.pl

**Keywords:** collagen glycosylation, *COLGALT2* gene, GTL25D2 protein, in silico prediction tools, rectal prolapse, Sanger sequencing, whole-exome sequencing

## Abstract

Rectal prolapse is influenced by many factors including connective tissue dysfunction. Currently, there is no data about genetic contribution in the etiology of this disorder. In this study, we performed trio whole-exome sequencing in an 11-year-old girl with mucosal rectal prolapse and her parents and sibling. Genetic testing revealed a novel heterozygous missense variant c.1406G>T; p.G469V in exon 11 of the *COLGALT2* gene encoding the GLT25 D2 enzyme. Sanger sequencing confirmed this variant only in the patient while the mother, father and sister showed a wild-type sequence. The pathogenicity of the novel variant was predicted using 10 different in silico tools that classified it as pathogenic. Further, in silico prediction, according to Phyre2, Project HOPE, I-Mutant3.0 and MutPred2 showed that the missense variant can decrease protein stability and cause alterations in the physical properties of amino acids resulting in structural and functional changes of the GLT25D2 protein. In conclusion, the present study identifies a previously unknown missense mutation in the *COLGALT2* gene that encodes the enzyme involved in collagen glycosylation. The clinical features observed in the patient and the results of in silico analysis suggest that the new genetic variant can be pathogenic.

## 1. Introduction

Rectal prolapse (RP) is a condition that causes the extrusion of some or all of the rectal wall through the anal orifice. Pediatric rectal prolapse usually occurs prior to 4 years of age with equal frequency in boys and girls [1]. However, the highest incidence of this disorder has been noted in the first year of life. There are two types of RP: type I–true procidentia, or complete prolapse (full-thickness extrusion of the rectal wall) and type II–false procidentia, partial or mucosal prolapse (only mucosa protruded, the most common in children before the age of 2) [2]. Rectal prolapse is rare in children without predisposing conditions such as chronic constipation or chronic diarrhea as well as cystic fibrosis, Ehler-Danlos syndrome and Hirschsprung’s disease. In children with RP, anatomical features, such as vertical course of the rectum, pelvic floor defects or deep Douglas pouch, should also be considered. In children, prolapse of the rectal mucosa occurs when the mucosa is poorly attached to the submucosa, which may be caused by loosening and stretching of connective tissue attachments. The prolapse of the rectal mucosa can be caused by the loosening and stretching of connective tissue attachments, so the mucosa is poorly attached to the submucosa. Additionally, abnormal connective tissue function can lead to pelvic floor descent and eventually pelvic organ prolapse (POP) [3].

**Figure 1:**
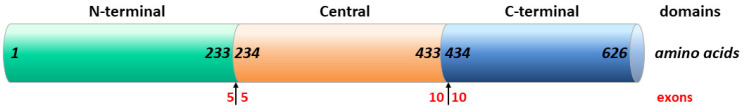
Schematic diagram of the domains within GLT25D2 protein. N-terminal domain is not involved in enzymatic activity. Central and C-terminal domains are important for enzyme activity. C-terminal domain is also involved in the recognition of donor and acceptor substrates [8].

Collagens are the main component of connective tissue, undergoing post-translational modification including hydroxylation (proline and lysine residues) and glycosylation of hydroxylysine residues (HyK) [4,5]. Both lysine hydroxylation and glycosylation are important for the assembly of collagen molecules, cell–matrix interactions and signaling [6]. Defective collagen hydroxylation underlies various diseases, such as Ehlers-Danlos and Bruck syndrome. The role of collagen glycosylation in the pathophysiology of collagen disorders remains elusive because the disease associated with the dysfunction of this process is not known. Two collagen galactosyltransferases COLGALT1 (GLT25D1) and COLGALT2 (GLT25D2) catalyze the first step of O-glycosylation of collagen HyK residues. GLT25D2 enzyme is not broadly expressed across tissues as GLT25D1; its expression is relatively high in the brain, muscle tissue and gastrointestinal tract compared to other tissues/organs (www.proteinatlas.org, 31 January 2022) (Supplementary Figure S1). Human GLT25D2 is a 626-amino acid protein and is encoded by the *COLGALT2* gene located on chromosome 1q25.3. The *COLGALT2* gene consists of 13 exons, the GT25 domain required for galactosyltransferase activity being encoded by exons 7 to 12 [7]. The schematic structure of the GLT25D2 protein is shown in Figure 1. The prediction of protein secondary structure and motifs crucial for enzymatic activity are detailed in Supplementary Figure S2.

In the present study, we have explored the potential molecular changes underlying mucosal rectal prolapse in the proband. To the best of our knowledge, this is the first study in which a trio-based whole-exome sequencing (WES) approach was used. Our study has revealed a novel mutation, c.1406G>T (p.G469V), in the *COLTGALT2* gene that seems to affect the patient’s phenotype. However, to determine whether the mutation actually affects the enzyme activity, a functional assessment is required.

## 2. Results

### 2.1. Clinical Findings

**Figure 2:**
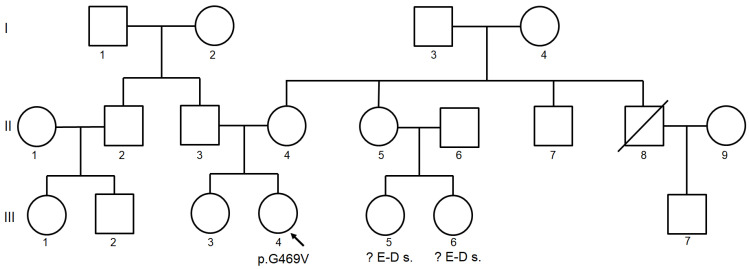
Three-generation family pedigree of the proband. The proband carries a novel variant p.G469V in the *COLGALT2* gene. E-Ds–Ehlers-Danlos syndrome was excluded after genetic examination.

The three-generation pedigree was provided based on the patient family history (Figure 2). The patient’s parents (II-3 and II-4) and sister (III-3) were healthy. In relatives III-5 and III-6, Ehlers-Danlos syndrome (EDS) type III was suspected on the basis of clinical features, however, the genetic test for EDS was negative.

MRI showed prolapse of the rectum mucosa (15 mm), slightly increasing when the patient tried to push. Also, the degree of vaginal prolapse increased when the patient tried to push. Furthermore, vascular changes that were noticed during the first MRI study were confirmed. Additionally, a slight backward bulging of the intervertebral discs was observed, probably resulting from the laxity of the ligamentous apparatus.

### 2.2. Identification of COLGALT2 Variant

**Figure 3:**
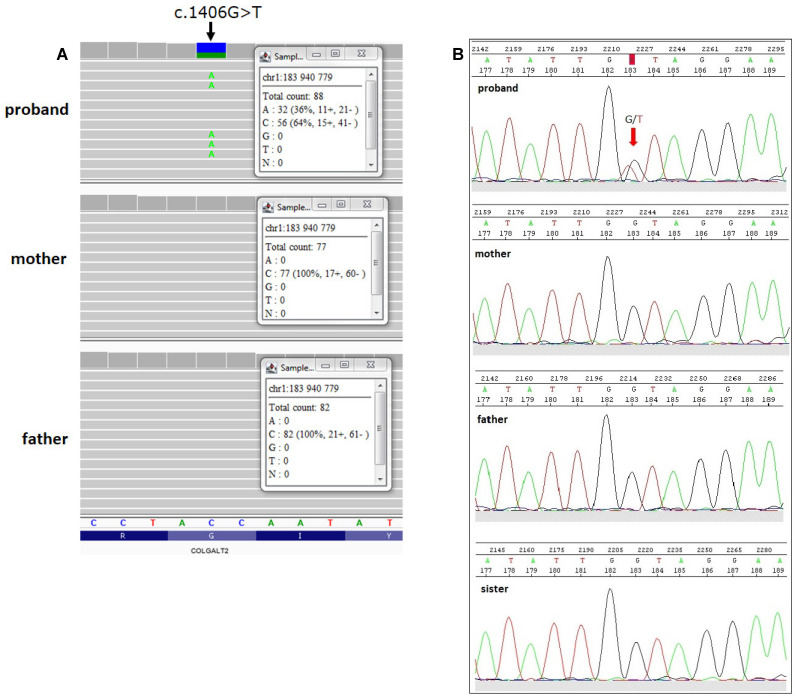
Identification of a novel missense mutation in the *COLGALT2* gene. (**A**) Trio WES results of the c.1406G>T (p.G469V) variant. Integrative Genomic Viewer screenshot. Coverage and numbers of reads indicated in inserts. (**B**) Sanger sequencing in the proband’s family. The proband is a carrier of heterozygous missense variant in exon 11, c.1406G>T resulting in amino acid substitution G469V. Her mother, father and sister did not carry this variant.

Genetic studies revealed a heterozygous missense variant in the *COLGALT2* gene which was absent in the proband’s parents (hg38: chr1: g.183940779-C>A, NM_015101.4: c.1406G>T, p.G469V) (Figure 3A). In GnomAD database the studied variant has an allele frequency of 0. The same allele frequency of the p.G469V variant was annotated in an in-house database of >5000 WES of Polish individuals. Based on the 10 pathogenic predictions from BayesDel_addAF, DANN, EIGEN, FATHMM-MKL, LIST-S2, M-CAP, MVP, MutationAssessor, SIFT and MutationTaster, the p.G469V variant was classified as pathogenic or deleterious. According to the CADD predictor, the studied variant had a score value of 33.

To validate the NGS variant, Sanger sequencing was performed in the proband, proband’s parents and sister. As a result, a heterozygous variant c.1406G>T was confirmed in exon 11 of the *COLGALT2* gene in the proband and should be considered as a *de novo* variant. Sanger sequencing of her parents and sister showed a wild-type (Figure 3B).

### 2.3. In Silico Assessment of Protein Structure, Stability and Interactions

**Figure 4:**
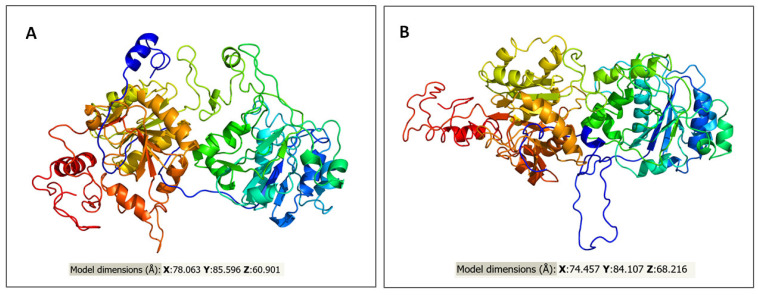
Predictive 3D models for GLT25D2 protein. (**A**) Predictive 3D structural model of normal GLT25D2 protein. (**B**) Predictive 3D structural model of the G469V variant. The images are rainbow colored from N–terminus to C–terminus.

We used a Phyre2 homology modelling tool to generate the three-dimensional structure of the GLT25D2 protein. This in silico approach revealed that the model dimension (Å) of the native and G469V protein differ mainly in the value of the X and Z axes. The predictive 3D models of both proteins are presented in Figure 4.

**Figure 5:**
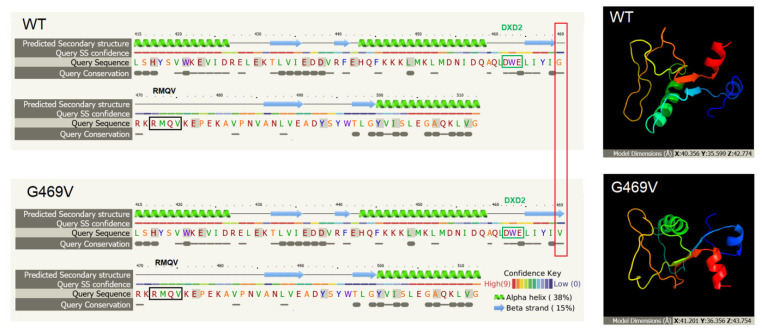
Predicted secondary structure and residues conservation of wild-type and mutant GLT25D2 proteins encoded by exons 10 and 11 of the *COLGALT2* gene. The thin grey bar indicates moderate conservation and a large block indicates a high degree of conservation. The red rectangle shows the position of the amino acid substitution. WT–wild-type protein, G469V–glycine to valine substitution at amino acid position 469. DXD2 and RMQV–motifs important for enzymatic activity. The 3D models of GLT25D2 domain (415 to 512 aa) are colored in a rainbow from N terminus to C terminus.

Furthermore, Phyre2 analysis showed that substituted amino acid is located near a highly conserved protein region (Figure 5).

Project HOPE tool was used to analyze the structural and physicochemical properties of the amino acid substitution of the protein variant [9]. This analysis revealed that the original wild-type residue (glycine) and mutant residue (valine) differed in size, charge and hydrophobicity-value. The mutant residue was bigger and more hydrophobic than the wild-type residue. Moreover, the wild-type glycine residue was the most flexible of all residues, flexibility being necessary for the protein’s function; therefore, mutation can/could abolish it. Project HOPE also confirmed the Phyre2 prediction that the mutant residue was located near a highly conserved position and the conservation score indicated that this mutation was probably damaging to the protein. MutPred2 results showed the probability of a deleterious mutation score for G469V of 0.691 and the *p*-Value lower than <0.05, indicating that substitution can alter the structural and functional properties of the GLT25D2. Thus, MutPred2 analysis reconfirmed the Project HOPE predictions that amino acid substitution could affect protein function.

The prediction of the effect of the G469V variant on the protein stability was performed with the I-Mutant3.0 software. The results were as follows: G469V variant decrease protein stability with reliability index 7 and the change value of free energy (DDG) equals –0.49 Kcal/mol, (DDG = DG (new protein)–DG (wild-type) in Kcal/mol). Stability change was calculated at pH 7 and 25 #xB0;C.

**Figure 6:**
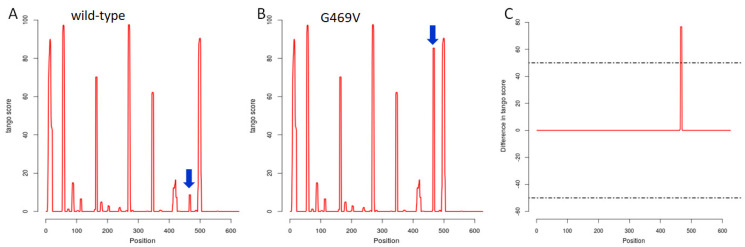
TAGO prediction by SNPeffect 4.0. (**A**,**B**) Profile representation of the TANGO stretches in the wild-type (WT) and G469V variant. (**C**) Difference in TANGO aggregation between WT and G469V variant. The flat line indicates that the variant does not alter the aggregation profile of the protein. Positive peaks indicate increased aggregation tendency due to the G469V variant. From left to right, all residue scores from the N–terminus to the C–terminus are plotted.

**Figure 7:**
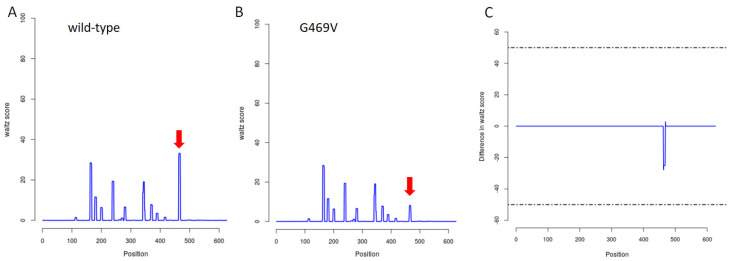
WALZ prediction by SNPeffect 4.0. (**A**,**B**) Profile representation of the WALZ stretches in the wild-type (WT) and G469V variant. (**C**) Difference in WALZ aggregation between WT and G469V variant. Negative peaks indicate decreased amyloid propensity due to the G469V variant. From left to right, all residue scores from the N–terminus to the C–terminus are plotted.

In order to predict the effect of the G469V variant on the structural phenotype of the GLT25D2, the SNPeffect 4.0 database was applied. The results obtained from the TAGO algorithm meant that the mutation increased the aggregation tendency of GLT25D2 (dTANGO = 385.31). The TANGO aggregation score and difference in TANGO aggregation of the wild-type and G469V variant are shown in Figure 6. In turn, WALZ algorithm predicted that the G469V variant decreased the amyloid propensity of the protein (dWALTZ = −150.39) (Figure 7). However, the G469V variant does not affect the chaperone binding tendency of the GLT25D2 protein as predicted by LIMBO (dLIMBO = 0.00).

**Figure 8:**
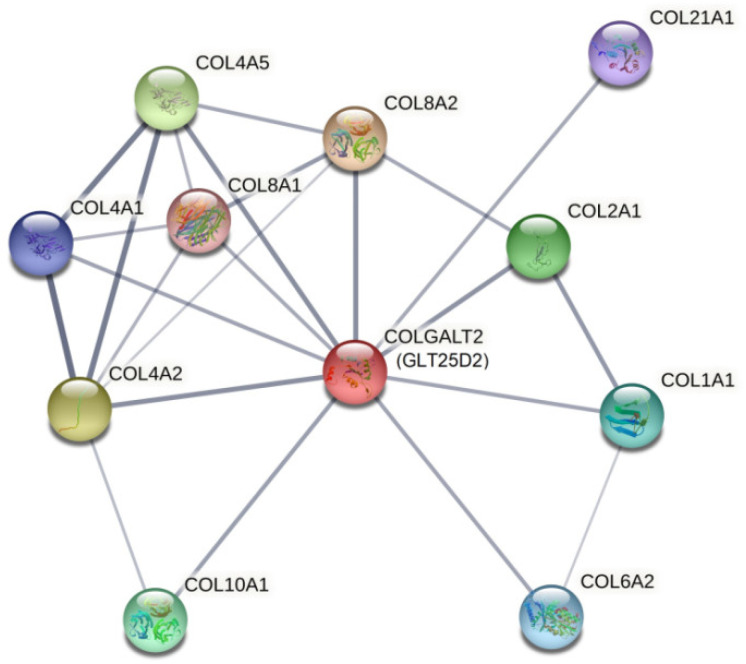
The protein-protein interaction network of the GLT25D2 (COLGALT2) protein. The diagram presents only physical subnetworks based on confident data performed by STRING. The thickest edge between proteins indicates the highest confidence in protein-protein association. Minimum required interaction score: 0.400; the number of interactions to show: 10; average node degree: 4.18; average local clustering coefficient: 0.799; Protein-protein interactions (PPI) enrichment *p*-value: <0.001.

Protein-protein interactions are crucial for most cellular processes; therefore, the identification of the interaction network was carried out by the STRING database. STRING interaction analysis revealed that native GLT25D2 protein interacts with various types of collagen, including collagen type IV, II and VIII (Figure 8).

## 3. Discussion

This study reported a novel genetic variant of the *COLGALT2* gene (c.1406G>T) within exon 11, detected by WES and confirmed by Sanger sequencing. The *COLGALT2* gene encodes a collagen galactosyltransferase (ColGalT) enzyme (called GLT25D2). This point mutation resulted in a glycine to valine substitution at amino acid position 469 (p.G469V). This missense mutation was found as a heterozygous state in the proband with mucosal rectal prolapse and confirmed as a de novo genetic variant because Sanger sequencing of her parents and sister showed a wild-type (Figure 3B). This variant was absent from databases such as the GnomAD database and the Polish in-house WES database (database of >5000 WES). Interestingly, the G469V variant was classified as pathogenic by all prediction programs: BayesDel_addAF, DANN, EIGEN, FATHMM-MKL, LIST-S2, M-CAP, MVP, SIFT, MutationAssessor and MutationTaster. In silico analysis, with Phyre2 and Project HOPE, demonstrated that amino acid substitution occurred near a highly conserved region. Since protein conformation is crucial for enzymatic activity, we performed further in silico analyses to identify the effect of mutation on protein structure, function and/or stability. Both Project HOPE and MutPred2 servers predicted that this variant was deleterious and negatively affected the structure of the protein. Interestingly, the position of amino acid substitution (glycine to valine) identified in the present study was located between two motifs, DXD and RMQV essential for the enzymatic activity (Figure 5). Valine (Val) and glycine (Gly) residues have different properties; Val is bigger and more hydrophobic than Gly. Moreover, Gly is the most conserved and flexible residue, being flexible enough to make the torsion angles. It is plausible that the mutation introducing Val can disturb the local structure and conformational flexibility of the GLT25D2 protein. It is worth underlining that glycine residues may provide flexibility necessary for enzyme active sites to induce conformational changes [10]. Likewise, the stability of the tertiary structure of the protein is also believed to be relevant for maintaining the function of the protein [11]. By using the I-Mutant3.0 tool, we established that the G469V variant decreased the GLT25D2 protein stability, which may have impacted its enzymatic activity.

The enzyme GLT25D2 is one of two members of the galactosyltransferase family (GLT25D1 and GLT25D2) that transfer galactose to HyK residues in mannose binding lectin and collagen [12]. In addition to hydroxylation, collagen glycosylation is an important post-translational modification prior to the formation of the collagen triple helix and takes place in the endoplasmic reticulum [4]. The degree of collagen glycosylation varies among different collagen types and tissues. Glycosylation is more extensive in less-organized collagen type IV than in fibrillar collagens including types I, II or III [13]. Type IV collagen, the main constituent of the basement membrane, forms a network-like structure in the extracellular matrix providing a scaffold to many other macromolecules [14]. This network makes up a large portion of the basement membrane zone and underlies epithelial and endothelial cells [15]. Type IV collagen network provides mechanical support and confers the tensile strength of the basement membrane as well as acts as a substrate to which cells adhere [16,17,18]. In fact, one study has shown that glycosylated HyKs are necessary for the correct localization of type IV collagen in the basement membrane [19]. Furthermore, this study provided evidence that glycosylation of HyKs is crucial for the secretion and tetramerization of type IV collagen, as well as the modification needed for the assembly of type IV collagen tetramers. A subsequent in vitro study demonstrated a novel functional role of collagen glycosylation. The experiments showed that it played an important role in collagen turnover, thus it is associated with connective tissue remodeling [20]. Due to the biological significance of collagen IV glycosylation, it should be taken into account in the etiology of connective tissue disorders. Interestingly, our protein-protein interaction analysis performed by STRING showed that the enzyme strongly interacted with COL4A2 and COL4A5 and, to a lesser degree, with COL4A4. COL4A2 is widely distributed in all basement membranes. In turn, COL4A5 is co-localized with COL4A4 within the skin, brain and gastrointestinal tract [21]. In addition to this, Kendall et al., using immunochemical staining methods, established that type IV collagen is present in the basement membrane of the thyroid gland [22].

Considering the above and clinical features of our patient (i.a., Hashimoto’s disease), we hypothesized that the patient’s phenotype might be affected by the G469V variant of the GLT25D2 protein. Interestingly, Hennet postulated that collagen glycosylation is probably related to the etiology of autoimmune disorders [4], which may correspond to the Hashimoto’s disease in our patient. It is plausible that insufficient collagen glycosylation disturbed collagen oligomerization affecting the structure of the extracellular matrix and the structural supporting function of the basement membrane. Therefore, changes in the composition and/or structure of the basement membrane may have resulted in the loosening of the attachment of the mucosa to the submucosa, causing mucosal rectal prolapse. We suggest, on the one hand, that the mild phenotype might arise from the heterozygous condition of the mutant enzyme and, on the other hand, a possible compensatory effect by *COLGALT1* expression. To our knowledge to date, there is limited evidence regarding the genetic variants of the *COLGALT2* gene associated with human connective tissue disorders. Surprisingly, only one study characterized the relationship between the SNP variant of the *COLGALT2* gene, impacting DNA methylation, and the etiology of osteoarthritis [23]. Moreover, two unrelated patients with cerebral small vessel abnormalities who carried biallelic variants in the *COLGALT1* gene, encoding the GLT25D1 enzyme, have been described [24]. Since literature data are limited and no model organism carrying defective collagen glycosylation has been described, it cannot be unequivocally concluded that the mutation had a detrimental effect.

## 4. Materials and Methods

### 4.1. Patient

**Figure 9:**
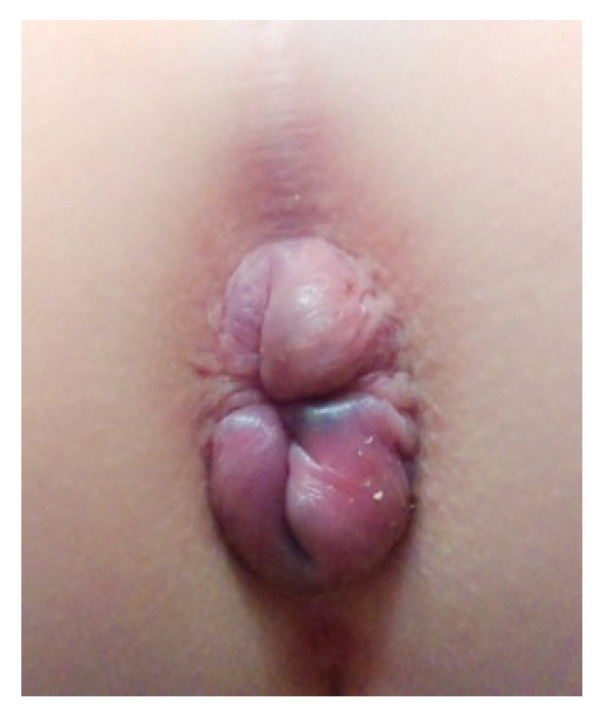
Mucosal rectal prolapse in the patient at the age of 9.

The proband was an 11-year-old girl referred to the Department of Gynecology and Obstetrics for gynecological consultation. The patient was the second of two siblings born from healthy non-consanguineous parents. She was born at full term with a birth weight of 3640 g, a height of 56 cm and haemangioma on the left thigh. At the age of about 7 months, mucosal rectal prolapse was noticed. The infant’s psychomotor development was normal. At the age of 8, the dynamic contrast-enhanced magnetic resonance imaging (DCE-MRI) of the pelvis and genetic consultation were carried out in this girl. DCE-MRI revealed dilation of the internal iliac artery, abnormal vessels at L5-S1 level on the left side and abnormal vessels below the ossification nucleus of the greater trochanter of the left femur. Based on the clinical features (i.e., joint pain, tendency to bruise and prolonged wound healing) and laboratory findings, Ehlers-Danlos syndrome and cystic fibrosis were excluded. The analysis of cytogenetic microarrays (Agilent ISCA 8 × 60K v2, Agilent Technologies Inc, Santa Clara, USA) showed no genetic changes essential for the functioning of connective tissue. At the age of 9, she was diagnosed with Hashimoto’s disease and at the age of 11 she was examined for celiac disease. However, considering mucosal rectal prolapse (Figure 9), pelvic organ prolapse (POP) associated with connective tissue dysfunction was strongly suggested. Therefore, the patient required a gynecological consultation. After gynecological consultation in our Department of Gynecology and Obstetrics, MRI of the pelvis and whole exome sequencing (WES) were carried out. This study was approved by the Bioethics Committee of the Andrzej Frycz Modrzewski Krakow University No KBKA/21/O/2019.

### 4.2. DNA Extraction

Genetic analysis was conducted in the proband, her parents and sister. This family provided written informed consent for genetic testing. Genomic DNA (gDNA) was prepared from peripheral blood using the QIAamp DNA Blood Mini Kit (Qiagen, Hilden, Germany), according to the manufacturer’s protocol. The quantity and quality of gDNA were assessed by a NanoDrop One spectrophotometer (Thermo Fisher Scientific, Inc., Waltham, MA, USA) and by agarose gel electrophoresis. Additionally, before the WES procedure, DNA quantification was carried out using a Qubit dsDNA HS assay kit (Invitrogen by Thermo Fisher Scientific, Inc., Waltham, MA, USA) and Qubit 4.0 Fluorometer (Invitrogen by Thermo Fisher Scientific, Inc., Waltham, MA, USA) following the manufacturer’s protocol.

### 4.3. WES Sequencing

Genetic study in the proband and her parents’ samples was performed using NGS-based whole exome sequencing (WES) using Human Core Exome Kit (Twist Bioscience, South San Francisco, CA, USA), according to the manufacturer’s instruction. Enriched library was paired-end sequenced (2 × 100 bp) on NovaSeq 6000 (Illumina, San Diego, CA, USA) to the mean depth of 145×. Bioinformatics analysis of raw WES data and variants prioritization were performed as previously described [25]. Finally, variants were inspected with Integrative Genomics Viewer (IGV).

### 4.4. Validation by Sanger Sequencing

The flanking region of the *COLGALT2* variant was amplified by PCR with primer pairs (F: 5′–CTAGCAAAATGGCCCCAGTG–3′ and R: 5′–AGTTCAGGGAGACTCACACG–3′) designed using the Primer 3 algorithm (https://primer3.ut.ee/, 15 March 2021). PCR was performed in a final volume of 20 μL containing PCRBIO Taq Mix Red (PCR Biosystems, London, UK) and 0.5 μL of each primer with a concentration of 10 μM (Genomed, Warsaw, Poland) using Thermocycler Eppendorf Mastercycler.

Amplicons were purified with QIAquick PCR Purification Kit (QIAGEN, Hilden, Germany). Purified PCR products were sequenced for both sense and antisense strands using the BigDye Terminator v1.1 Cycle Sequencing Kit (Thermo Fisher Scientific, Waltham, MA, USA). Sequencing reactions were purified by ethanol/sodium acetate precipitation. Sequencing products were analyzed by capillary electrophoresis with the 3130 Genetic Analyzer (Life Technologies, Carlsbad, CA, USA) and Sequencing Analysis Software v5.2 (Life Technologies, Carlsbad, CA, USA). Sanger sequencing was performed on specimens obtained from the patient and her family (parents and sister).

### 4.5. In Silico Tools

#### 4.5.1. Prediction of Variant Impact on the Protein Structure, Function and Stability

The in silico websites and servers including Phyre2 (Protein Homology/analogY Recognition Engine), Project HOPE, MutPred2 and I-Mutant 3.0 were used. 3D models of the wild-type and G469V variant were generated by Phyre2. To understand the structural and functional effects of the amino acid substitution, Project HOPE and MutPred2 web-servers were used. I-Mutant3.0 was used to predict protein stability changes upon mutation.

#### 4.5.2. Molecular Phenotypic Characterization of Protein Variants

For mapping the effects of the G469V variant on the molecular phenotype of protein, the SNPeffect 4.0 bioinformatics tool was applied. The SNPeffect integrates aggregation prediction (TANGO), amyloid prediction (WALTZ), chaperone-binding prediction (LIMBO) and protein stability analysis (FoldX) for structural phenotyping [26].

#### 4.5.3. Prediction of Protein Interactions

The molecular interaction network of the GLT25D2 protein with other proteins was visualized using STRING (Search Tool for the Retrieval of Interacting Genes) database. The STRING database computationally predicted the strength of an interaction between proteins, including both physical interactions as well as functional associations [27].

## 5. Conclusions

To summarize, we identified a novel missense mutation (c.1406G>T) in exon 11 of the *COLGALT2* gene. This mutation caused a highly conserved amino acid (Gly → Val) substitution at position 469 of the GLT25D2 protein. In silico prediction tools classified this variant as pathogenic and deleterious for protein structure, function and stability. Additionally, this variant was located between two motifs essential for ColGalT activity. Considering the position of amino acid replacement, we postulated that this variant has the potential to impact the patient’s phenotype. However, additional studies are required to ultimately confirm genotype-phenotype relationships.

## Data Availability

Due to the privacy policy, data are available on request after approval of the patients.

## Supplementary Materials

The following supporting information can be downloaded at: https://www.mdpi.com/article/10.3390/ijms23073670/s1.
