# Supplementary material for: Identification and In Silico Characterization of a Novel COLGALT2 Gene Variant in a Child with Mucosal Rectal Prolapse
Source: Int J Mol Sci. 2022 Mar 27;23(7):3670. doi: 10.3390/ijms23073670 (PMC8999070; doi:10.3390/ijms23073670)
Supplement: Supplementary file 1 [file ijms-23-03670-s001.zip › ijms-1623184-supplementary.pdf]

Supplementary Materials

Identification and *in silico* characterization of a novel *COLGALT2* gene variant in a child with mucosal rectal prolapse.

Anna Sadakierska-Chudy[1], Paweł Szymanowski[2], Arleta Lebioda[3], Rafał Płoski[4]

[1] Department of Genetics, Faculty of Medicine and Health Sciences, Andrzej Frycz Modrzewski Krakow University, Krakow, Gustawa Herlinga-Grudzinskiego 1, 30-705 Krakow, Poland, asadakierska-chudy@afm.edu.pl

[2] Department of Gynecology and Obsterics, Faculty of Medicine and Health Sciences, Andrzej Frycz Modrzewski Krakow University, Gustawa Herlinga-Grudzinskiego 1, 30-705 Krakow, Poland, pszymanowski@afm.edu.pl

[3] Department of Molecular Techniques, Department of Forensic Medicine, Wroclaw Medical University, M. Curie-Sklodowskiej 52, 50-369 Wroclaw, Poland, arleta.lebioda@umw.edu.pl

[4] Department of Medical Genetics, Warsaw Medical University, Pawinskiego 3c, 02-106 Warsaw, Poland, rploski@wp.pl

*Correspondence: asadakierska-chudy@afm.edu.pl, tel. +48 122524590

Supplementary Figure S1 The tissue expression of the *COLGALT2* and *COLGALT1* gene and the GLT252 and GLT25D1 proteins, respectively. Images acquired from the Human Protein Atlas (www.proteinatlas.org).

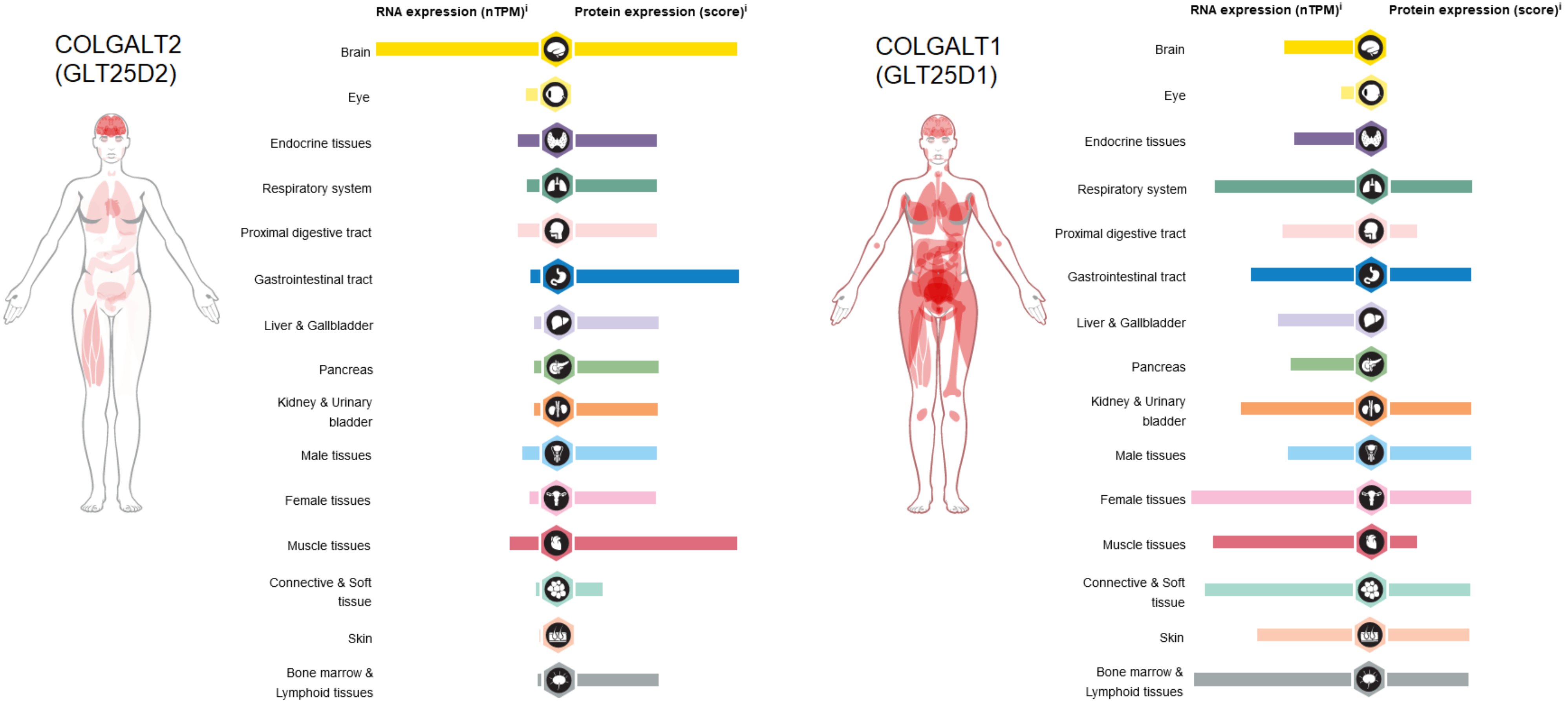

Supplementary Figure S2 The GLT252 protein secondary structure predicted by Phyre2. Motifs crucial for the enzymatic activity are marked according to previous analysis (Perrin-Tricaud et al., 2011).

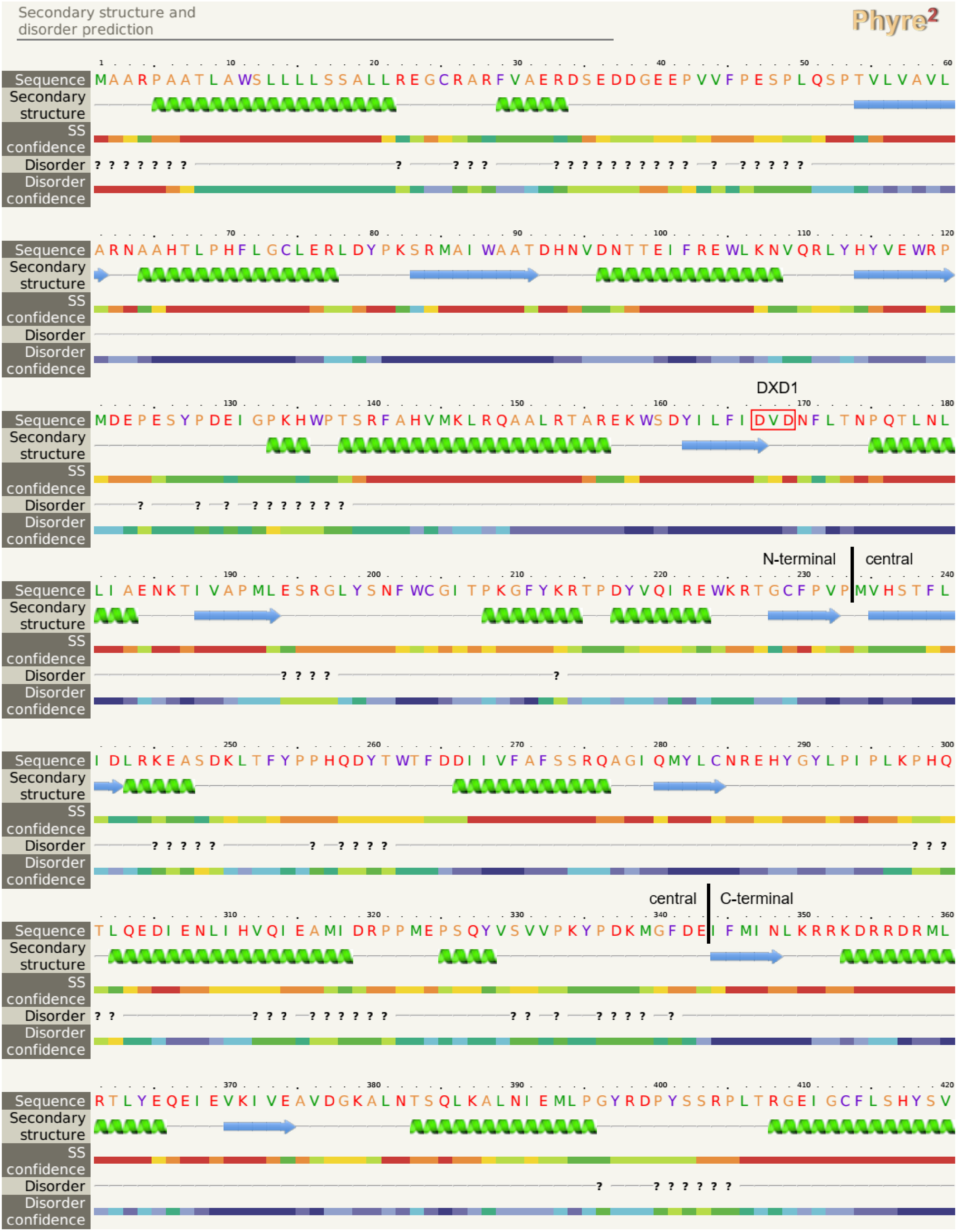

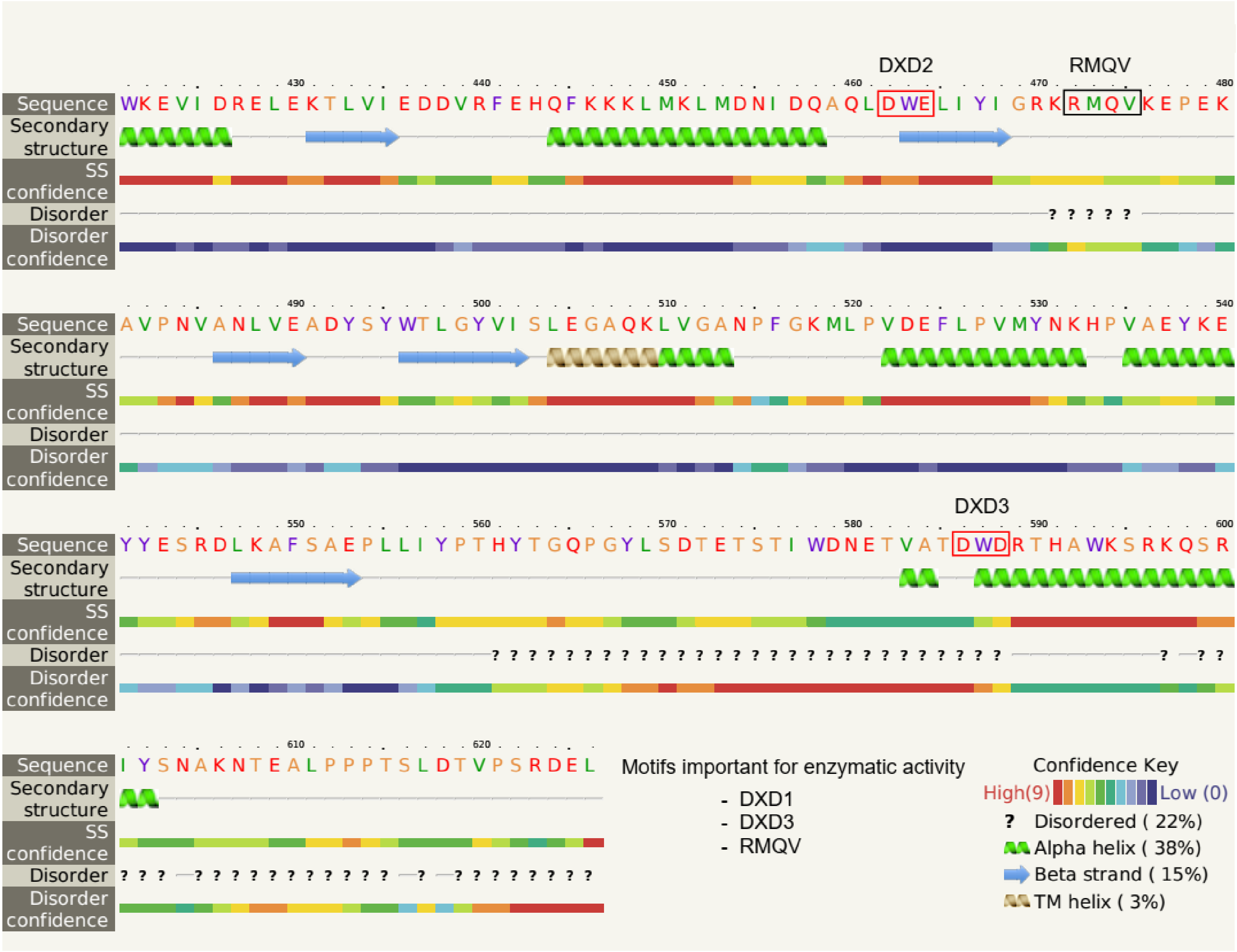

DXD2
RMQV
DXD3
Sequence
Secondary structure
SS confidence
Disorder
Disorder confidence
430 440 450 460 470 480
WKEVIDRELEKTLVIEDDVRFEHQFKKKLMKLMDNIDQAQLDWELIYIGRKRMQVKEPEK
490 500 510 520 530 540
AVPNVANLVEADYSYWTLGYVISLEGAQKLVGANPFGKMLPVDEFLPVMYNKHPVAEYKE
550 560 570 580 590 600
YYESRDLKAFSAEPLLIYPTHYTGQPGYLSDTETSTIWDNETVATDWDRTHAWKSRKQSR
610 620
IYSNAKNTEALPPPTSLDTVPSRDEL
Motifs important for enzymatic activity
- DXD1
- DXD3
- RMQV
Confidence Key
High(9)
Low (0)
? Disordered (22%)
Alpha helix (38%)
Beta strand (15%)
TM helix (3%)
